# Diseases of the Digestive Organs in Infancy and Childhood

**Published:** 1886-10

**Authors:** 


### BOOK REVIEWS.

DISEASES OF THE DIGESTIVE ORGANS IN INFANCY AND CHILDHOOD, *with Chapters on the Investigation of Disease and on the General Management of Children. By* LOUIS STARR, M. D., *Clinical Professor of Diseases of Children in the Hospital of the University of Pennsylvania, Physician to the Children's Hospital, Philadelphia, etc. With colored plates and other illustrations. Philadelphia:* P. BLAKISTON SON & CO. *Chicago:* W. T. KEENER. *1886.*

Dr. Starr's latest work, like his other writings, is preeminently practical and interesting. Its object, as set forth in the preface, "is to give prominence to a class of disorders constituting a large proportion of the ailments of childhood, but often too briefly considered in works on pædiatrics."

The book is exactly what it professes to be, viz., a monograph on the buccal, gastric, and intestinal lesions of children, no unimportant part of their ailments. It opens with a discussion of the clinical investigation of the diseases of childhood in general, which, while it is good and comprehensive, embraces very little that is new, except, perhaps, the hints in the sections on methods of drinking observed in children.

Section second opens the consideration of the diseases of the digestive tract with those of the mouth. More is here made of catarrhal stomatitis than the experience of the majority of general practitioners would justify. In fact, Dr. Bush would probably claim Prof. Starr's catarrhal stomatitis was really his œsophagitis under another name; and Dr. Starr's treatment really looks that way, as it is apparently directed more towards the gastric than to the buccal mucous membrane. Aphthous stomatitis evidently is considered to have the same ætiology, for it is similarly dealt with, and chlorate of potash, which in these cases is almost a specific, is only spoken of as a local application. On the other hand, it is highly recommended in ulcerative stomatitis, where, unfortunately, it generally disappoints, whether given internally or locally applied. Perhaps the fresh air and stimulants so highly spoken of in the treatment of these cases may have had much to do with the success of the potassium chlorate treatment, which under other surroundings would have failed. Noma, thrush and the sympathetic troubles of difficult dentition conclude the diseases of the mouth and present nothing new or of especial interest in either theory or practice. The affections of the throat discussed are simple pharyngitis, catarrh of the tonsils, follicular tonsilitis, abscess and hypertrophy of the tonsils and retropharyngeal abscess. Unlike Professor Pepper, he regards follicular tonsilitis as always favorable in prognosis, and only by the most inexperienced to be confounded with diphtheria.

There certainly is very little resemblance between the irregular follicular deposits of the first and typical diphtheritic membrane; and yet it ought always to be kept in mind that about these innocent, cheesy dots diphtheritic membrane may and often does form, and that, whether or no there is a specific virus about them, they not infrequently attack the members of a family in succession, even when they have not been exposed to what Dr. Starr considers their most efficient cause, viz., a children's party.

The section on the affections of the stomach and intestines is the best in the book; its special value being in the very great care taken in the preparation of special diet tables for particular forms of indigestion. Dietetic rules in these cases are too often so general that it is exceedingly satisfactory to find an author explicit in his directions as to exactly what and when the child shall be fed. This is what Dr. Starr does emphatically, whenever the question of diet is an important factor in the treatment of the case, and some of his diet tables, as that for mucous disease, are models of their kind and invaluable. In fact, the book might be justly entitled "The Dietetics of Children, Sick and Well," so largely does this feature appear—and wisely, too, we might add—for success or failure in treatment of a sick infant often turns on as seemingly an unimportant matter as a few inches of dirty rubber tubing in a lazy mother's nursing-bottle, to which, by the way, Dr. Starr pays his respects, and advises that in every case of a bottle-fed child the physician should insist upon seeing, himself, the bottle used in feeding. The work abounds in just such common-sense hints, and is both timely and helpful to all who have occasion to treat children. Its differential diagnoses are pre-eminently practical, and its pathology full enough for any one except the pedant who prefers its minutiæ to success in practice. At times scarcely care enough has been taken to make his mixtures palatable for children, and his use of stimulants is freer than would be pleasing to the Weeks-Burnett combination; but these are trifling defects, if any, and Dr. Starr's *Diseases of the Digestive Organs* is a valuable addition to the American literature of pædiatrics. M. P. H.

